# A New Cell Model for Parkinson's Disease

**DOI:** 10.1371/journal.pbio.0020385

**Published:** 2004-10-05

Clinical descriptions of Parkinson's disease remain remarkably similar to those first described by James Parkinson nearly 200 years ago. Patients with “shaking palsy” experience a progressive loss of muscle control, increased muscle rigidity, inhibited movement, and tremors. These symptoms, it was later discovered, result from the loss of dopamine-producing neurons specifically in an area of the ventral midbrain called the substantia nigra. Midbrain dopamine neurons relay chemical signals that regulate motor control and less quantifiable attributes like mood and motivation, and therefore the loss of these cells is predicted to lead to the symptoms of Parkinson's.

**Figure:**
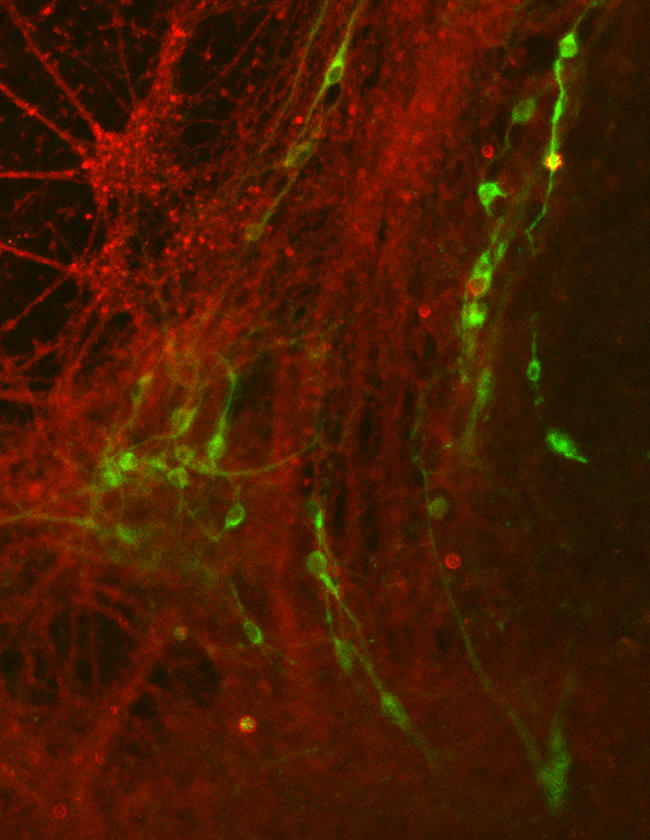


Despite the well-characterized cellular basis of Parkinson's disease, the molecular mechanisms responsible for dopamine neurodegeneration remain unknown. There is evidence that both genetic and environmental components are involved. That a person with Parkinson's disease is three to four times more likely than an unaffected individual to have a close family member with “parkinsonian” symptoms suggests a genetic factor; furthermore, several genes have been associated with relatively rare, familial forms of the disease. For example, mutations of the protein alpha-synuclein (α-synuclein), which is found to aggregate in the brains of patients with Parkinson's, lead to a familiar parkinsonism syndrome. Mutations in a second gene called DJ-1 were recently found in two families with an inherited form of Parkinson's. Importantly, mutations in DJ-1 have previously been linked to the pesticide paraquat in unrelated research on cell stress and reactive oxygen species, and have been linked to dopamine neuron toxicity. Reactive oxygen species are molecular byproducts of oxygen metabolism that react with and damage cellular components like proteins and DNA, and there is evidence from postmortem studies that reactive oxygen species may play a role in Parkinson's disease.

Part of the challenge of untangling the relative contributions of all these components stems from the difficulty in finding a model that can adequately mimic the loss of dopamine cells. In two papers published in *PLoS Biology*, Asa Abeliovich and colleagues make the case that a model based on mouse embryonic stem cells offers a promising platform for dissecting the disease mechanism of Parkinson's. Working with these cells, the researchers report that DJ-1-deficient cells—and especially DJ-1-deficient dopamine neurons—display heightened sensitivity to oxidative stress. In a second paper, they link DJ-1 dysfunction to alpha-synuclein aggregation.

Oxidative stress has long been associated with neuronal cell death and neurodegenerative diseases like Parkinson's. Proving a causal relationship between oxidative stress and neurodegeneration, however, requires establishing a molecular mechanism.

In the first paper, to explore the hypothesis that DJ-1 contributes to the cellular response to oxidative stress, Abeliovich and colleagues created mouse embryonic stem cells lacking functional copies of DJ-1 and exposed them to hydrogen peroxide, a powerful oxidizer. Compared to normal cells, DJ-1 mutants showed signs of greater toxicity and higher levels of cell death. These defects were corrected when the researchers reintroduced the protein in the mutants, confirming DJ-1's responsibility for the defects. DJ-1 protects against oxidative damage, the results show, not by inhibiting the accumulation of the reactive oxygen species associated with hydrogen peroxide, but by mitigating the damage created by them.

Abeliovich and colleagues then explored DJ-1's function in dopamine neurons by inducing mutant and control embryonic stem cells to differentiate in cell cultures. Production of dopamine neurons was significantly reduced in the DJ-1-deficient cultures relative to the control cultures. And like DJ-1-deficient embryonic stem cells, DJ-1 dopamine mutants were vulnerable to oxidative stress. “DJ-1 deficiency,” the authors conclude, “leads to reduced dopamine neuron survival and predisposes these cells to endogenous and exogenous insults.” Inhibiting DJ-1 activity in neurons from the embryonic mouse midbrain produced the same results.

In the second paper, Abeliovich and colleagues go on to probe the molecular basis of DJ-1's activity. There have been several leads regarding how DJ-1 functions, based on homology to related genes, including a potential role as a molecular protein chaperone; protein chaperones assist in the folding and refolding of damaged proteins, and thus play a central role in the cellular response to oxidative stress. Abeliovich and colleagues found that DJ-1 acts as an unusual molecular chaperone that is specifically induced under oxidative conditions, and acts to prevent the aggregation of cellular proteins. Interestingly, the researchers go on to show that one substrate of DJ-1 activity is alpha-synuclein, thus providing a possible mechanism linking these two molecules implicated in Parkinson's disease. Altogether, these results support a link between toxin-induced oxidative damage and disease, and provide a tractable model for studying the molecular mechanisms of neurodegenerative disease.

